# Supplementary material for: DIRAS3-Derived Peptide Inhibits Autophagy in Ovarian Cancer Cells by Binding to Beclin1
Source: Cancers (Basel). 2019 Apr 18;11(4):557. doi: 10.3390/cancers11040557 (PMC6521623; doi:10.3390/cancers11040557)
Supplement: Supplementary file 1 [file cancers-11-00557-s001.pdf]

Supplementary Material

DIRAS3-Derived Peptide Inhibits Autophagy in Ovarian Cancer Cells by Binding to Beclin1

Margie N. Sutton, Gilbert Y. Huang, Xiaowen Liang, Rajesh Sharma, Albert S. Reger, Weiqun Mao, Lan Pang, Philip J. Rask, Kwangkook Lee, Joshua P. Gray, Amy M. Hurwitz, Timothy Palzkill, Steven W. Millward, Choel Kim, Zhen Lu and Robert C. Bast Jr.

Sample Name :TatD3S2
Sample ID :U3505DB070-1

Pump A : 0.065% trifluoroacetic in 100% water (v/v)
Pump B : 0.05% trifluoroacetic in 100% acetonitrile (v/v)
Total Flow:1 ml/min
Wavelength:220 nm
<<LC Time Program>>

Time	Module	Command	Value
0.01	Pumps	Pump B Conc.	5
10.00	Pumps	Pump B Conc.	35
35.00	Pumps	Pump B Conc.	95
41.00	Pumps	Pump B Conc.	95
41.01	Pumps	Pump B Conc.	5
50.00	Pumps	Pump B Conc.	5
50.01	Controller	Stop	

<<Column Performance>>
<Detector A>
Column : AlltimaTM C18 4.6 x 250 mm

<Chromatogram>

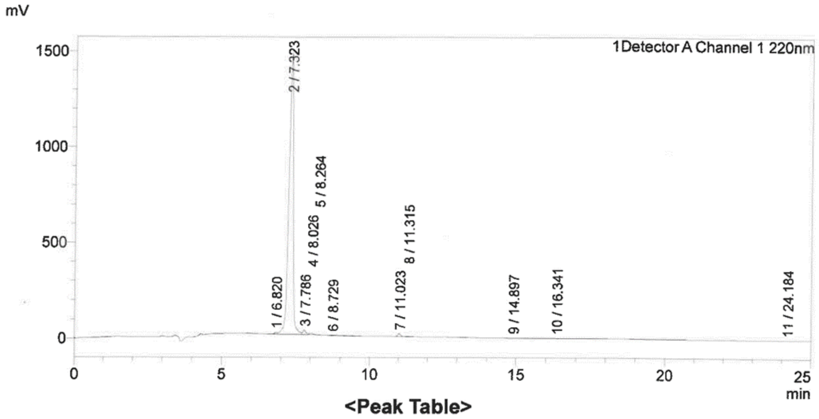

<Peak Table>

Detector A Channel 1 220nm

Peak#	Ret. Time	Area	Height	Area%
1	6.820	58302	9223	0.391
2	7.323	14475415	1471318	96.986
3	7.786	192222	23731	1.288
4	8.026	58633	7169	0.393
5	8.264	1273	275	0.009
6	8.729	16229	1699	0.109
7	11.023	98266	14343	0.658
8	11.315	16154	1925	0.108
9	14.897	1778	225	0.012
10	16.341	1178	69	0.008
11	24.184	5820	630	0.039
Total		14925272	1530609	100.000

Figure S1. Liquid chromatogram of Tat-D3S2 peptide synthesis.

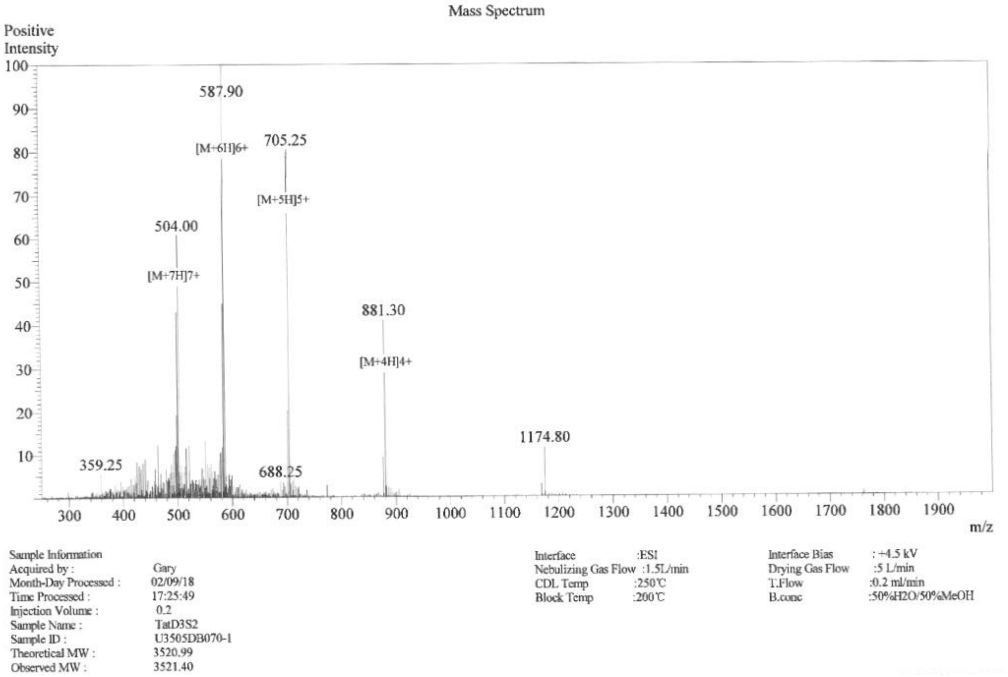

Figure S2. Mass spectrometry of Tat-D3S2 peptide synthesis.

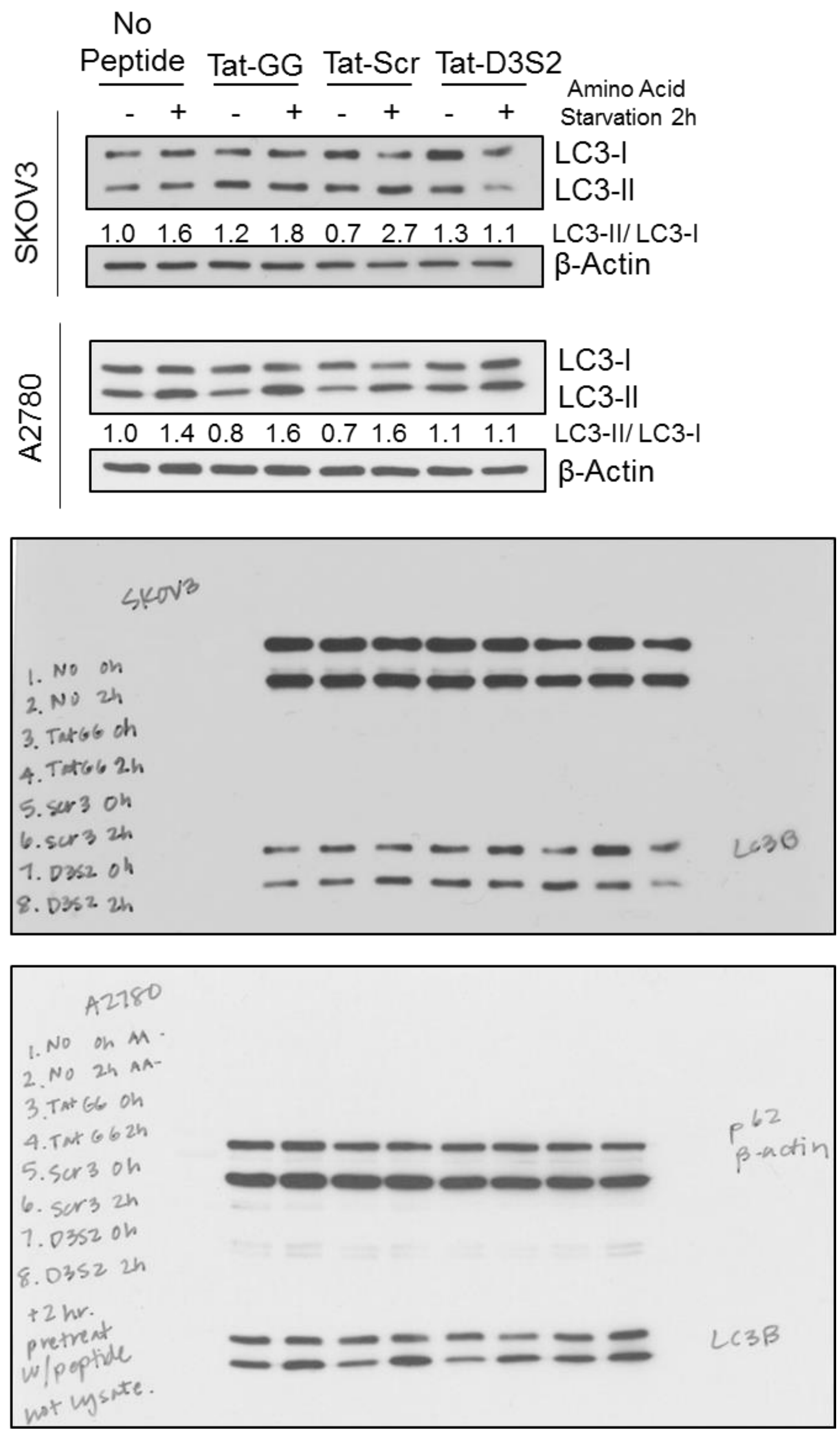

Figure S3. Raw data films for western blot analysis shown in Figure 5C.
